# Retrospective audit of 957 consecutive ^18^F-FDG PET–CT scans compared to CT and MRI in 493 patients with different histological subtypes of bone and soft tissue sarcoma

**DOI:** 10.1186/s13569-018-0095-9

**Published:** 2018-04-09

**Authors:** Ruth E. Macpherson, Sarah Pratap, Helen Tyrrell, Mehrdad Khonsari, Shaun Wilson, Max Gibbons, Duncan Whitwell, Henk Giele, Paul Critchley, Lucy Cogswell, Sally Trent, Nick Athanasou, Kevin M. Bradley, A. Bassim Hassan

**Affiliations:** 1 Oxford Sarcoma Service (OxSarc), Oxford University Hospitals Foundation Trust, Oxford, OX3 7LE UK; 2 Department of Radiology, Oxford University Hospitals Foundation Trust, Oxford, OX3 7LE UK; 3 Department of Oncology, Churchill Hospital, Oxford University Hospitals Foundation Trust, Oxford, OX3 7LE UK; 4 Nuffield Orthopaedic Centre, Oxford University Hospitals Foundation Trust, Oxford, OX3 7LE UK; 5 Department of Paediatric Oncology, Oxford University Hospitals Foundation Trust, Oxford, OX3 7LE UK; 6 NIHR Musculoskeletal Biomedical Research Unit (Sarcoma Theme), Sarcoma and TYA Unit of the NHS Oncology Department, and Sir William Dunn School of Pathology, University of Oxford, South Parks Road, Oxford, OX1 3RE UK

**Keywords:** Sarcoma, Positron emission tomography, Multi-disciplinary, Staging, Therapeutic response

## Abstract

**Background:**

The use of ^18^F-FDG PET–CT (PET–CT) is widespread in many cancer types compared to sarcoma. We report a large retrospective audit of PET–CT in bone and soft tissue sarcoma with varied grade in a single multi-disciplinary centre. We also sought to answer three questions. Firstly, the correlation between sarcoma sub-type and grade with ^18^FDG SUVmax, secondly, the practical uses of PET–CT in the clinical setting of staging (during initial diagnosis), restaging (new baseline prior to definitive intervention) and treatment response. Finally, we also attempted to evaluate the potential additional benefit of PET–CT over concurrent conventional CT and MRI.

**Methods:**

A total of 957 consecutive PET–CT scans were performed in a single supra-regional centre in 493 sarcoma patients (excluding GIST) between 2007 and 2014. We compared, PET–CT SUVmax values in relation to histology and FNCCC grading. We compared PET–CT findings relative to concurrent conventional imaging (MRI and CT) in staging, restaging and treatment responses.

**Results:**

High-grade (II/III) bone and soft tissue sarcoma correlated with high SUVmax, especially undifferentiated pleomorphic sarcoma, leiomyosarcoma, translocation induced sarcomas (Ewing, synovial, alveolar rhabdomyosarcoma), de-differentiated liposarcoma and osteosarcoma. Lower SUVmax values were observed in sarcomas of low histological grade (grade I), and in rare subtypes of intermediate grade soft tissue sarcoma (e.g. alveolar soft part sarcoma and solitary fibrous tumour). SUVmax variation was noted in malignant peripheral nerve sheath tumours, compared to the histologically benign plexiform neurofibroma, whereas PET–CT could clearly differentiate low from high-grade chondrosarcoma. We identified added utility of PET–CT in addition to MRI and CT in high-grade sarcoma of bone and soft tissues. An estimated 21% overall potential benefit was observed for PET–CT over CT/MRI, and in particular, in ‘upstaging’ of high-grade disease (from M0 to M1) where an additional 12% of cases were deemed M1 following PET–CT.

**Conclusions:**

PET–CT in high-grade bone and soft tissue sarcoma can add significant benefit to routine CT/MRI staging. Further prospective and multi-centre evaluation of PET–CT is warranted to determine the actual predictive value and cost-effectiveness of PET–CT in directing clinical management of clinically complex and heterogeneous high-grade sarcomas.

## Background

Conventional cross-sectional imaging techniques are routinely applied to the diagnostic and staging imaging of bone and soft tissue sarcomas, e.g. CT and MRI [1–3]. Diagnostic review of biopsies and staging scans in supra-regional multi-disciplinary teams (MDT), aims to inform clinical management in line with national and international sarcoma guidelines. MRI is also generally performed to guide local staging and restaging following neo-adjuvant therapy, whereas in subsequent post-treatment follow-up, high resolution CT is primarily used for the detection of distant metastases, particularly in the lung. As primary curative treatment for sarcoma is mostly surgical, accurate staging is essential in order to minimize inappropriate interventions in the presence of metastatic disease.

Combined ^18^F-FDG Positron Emission Tomography with CT (PET–CT) offers potential advantages with respect to sarcoma, as it provides both metabolic and anatomical imaging combined in a single examination. Sarcomas are frequently large tumours (> 5 cm) with intra-tumour regional and cellular heterogeneity, and frequently in larger tumours, central necrosis associated with hypoxia. As mesenchymal derived cancers, sarcomas may be detected by ^18^FDG uptake because of the general high metabolic activity and insulin sensitivity of these tissues. The maximum standardized uptake value (SUVmax) of ^18^FDG into higher-grade sarcoma appears to correlate with mitotic count and grade in some reported series [4, 5], and potentially with overall prognosis [6–10]. Reporting response assessment to oncological therapies using SUVmax before and after treatment, may also better correlate with histological response compared to dimensions alone [11]. With the advent of new systemic therapies that are either cytostatic or immune-modulatory, PET–CT has additional potential advantages in assessing responses depending on the therapeutic mechanism.

Despite these potential advantages, there have been relatively few reported series, with low cases numbers, providing information on which to base the routine application of functional PET–CT in specialist sarcoma clinical practice [12–20]. Moreover, in rare (< 6 per 100,000 population) sarcoma subtypes (approximately 80 different molecular based diagnostic subtypes in the WHO classification 2013), there appears even less ‘real world’ reported evidence for the application of PET–CT. Here, we retrospectively audit consecutively performed PET–CT scans in a supra-regional sarcoma centre. We initially attempted to address three questions; the correlation between histological sub-type and grade with ^18^FDG uptake; the use of PET–CT in the clinical setting of staging (during initial diagnosis), restaging (new baseline prior to definitive intervention) and treatment response, and the potential additional benefit obtained from PET–CT over concurrent conventional CT and MRI imaging in these settings.

## Methods

### Patient database

Retrospective audit of consecutive ^18^FDG PET–CT scans from one supra-regional UK sarcoma centre (Multi-disciplinary team, Oxford Sarcoma Service, Oxford University Hospitals Foundation Trust, UK) occurred between 1st February 2007 and 25th June 2014. The radiology search for scans was independently cross-referenced with the Oxford Sarcoma Service (OxSarc) sarcoma database (2006–2014) for diagnosis and outcome, to ensure that patients had been captured on both systems. The histological subtype and grade of each sarcoma was confirmed by pathology review and accessed via the local EPR system. Grade I sarcoma were regarded as low-grade, and grade II and III sarcoma as high-grade. All imaging (MRI, CT and PET–CT) were performed as directed either by the MDT, or were performed just prior to referral by an external clinician, with the information obtained used for consensus MDT agreement for subsequent clinical management. Thus, all scans performed and reported here were approved by the MDT specialist sarcoma team, and were therefore driven either as a result of the initial histology result of the biopsy, or appearances of local imaging of the presenting mass etc.

### Imaging procedures

PET–CT imaging from June 2007 to 2nd November 2009 was performed on a GE Discovery (STE BGO 16 slice CT, 400 MBq ^18^FDG, 60 min uptake period, fixed 80 mA/140 kV for CT, 4 min per bed position) and from 3rd November 2009, on a GE Discovery 690 (LYSO time of flight) 64 slice CT, 4 MBq/kg ^18^FDG, 90 min uptake period, modulated mA based on noise index (n = 25) 120 kV, 4 min per bed position). All scans included the skull base to either the upper thigh, or at least the joint below primary disease in the lower limbs where required, and included the head in skull or head and neck disease. Patients were fasted with a standard procedure for at least 6 h prior to the PET–CT. All the PET–CT examinations were independently reported by two consultant radiologists subspecializing in nuclear medicine and PET–CT, and were re-reviewed by a post CCT radiologist subspecializing in nuclear medicine/PET (RM) as part of this evaluation alongside the verified reports.

For the purpose of assessing the correlation between sarcoma grade and disease avidity, the FDG avidity calculated as standardized uptake value (SUVmax) on each PET–CT was recorded. SUVmax is simply the decay corrected maximum tracer activity (^18^FDG) within a volume of interest (VOI) and is available on all PET–CT scans. The standardized uptake value is (SUV) = [VOI activity concentration]/[injected activity/Weight g/mL], and if 1 mL of tissue volume is taken to weigh 1 g, then it becomes unit-less. The most FDG avid focus site of disease, whether primary or metastatic, was utilized as the SUVmax. In those cases where the SUVmax was not documented, this was re-calculated using standard GE supplied PET–CT analysis software. If there was CT evidence of a disease site however, with the FDG uptake at or below background (mediastinal blood pool) levels i.e. sites that are FDG essentially negative, the FDG avidity was documented as equal to 1.

### Imaging evaluation

All ^18^FDG PET–CT and conventional imaging modalities (CT and MRI) were re-analyzed with respect to sarcoma diagnostic sub-type, the timing and purpose of the scan with respect to staging, restaging or treatment response. The differences in sarcoma disease distribution between the ^18^FDG PET–CT and conventional MRI/CT scans were documented if scans were performed within 4 weeks of each other, otherwise any differences were not reported for the purpose of this evaluation. Not all patients had all types of scan within 4 weeks of each other, and so comparisons are necessarily retrospective and cannot be formally evaluated beyond descriptive reporting. For bone and pulmonary metastatic sites that were identified by PET–CT, the non-contrast enhanced CT components of the scan were examined separately and considered as the ‘conventional’ CT imaging modality. For soft tissue sites, including abdominal and pelvic sites, comparisons were made between ^18^FDG PET–CT and either separate contrast enhanced CT scans or MRI scans where possible. The specific ^18^FDG PET–CT advantages over conventional imaging were also determined for occult and solitary disease in either visceral, bone, muscular, sub-cutaneous and nodal sites. Histopathology was undertaken using standard diagnostic pathway with UK sarcoma reference pathologist (NA), using conventional WHO international guidelines (2012) and French based staging system (FNCCC). For the assessment of additional potential advantages of PET–CT, this was only conferred once histological data was reviewed to determine whether ‘PET positive’ disease had been histologically proven, or, if sampling had not been undertaken, where there was imaging evidence of progression over time confirming the presence of malignant disease, except for the common incidental unrelated sites in the colon, thyroid, prostate and breast.

### Statistical analysis

Descriptive statistics using Prism 7.0a were applied, including ANOVA and non-parametric Mann–Whitney tests. Sensitivity, specificity, positive predictive value and negative predictive values were calculated by standard means.

## Results

**Fig. 1:**
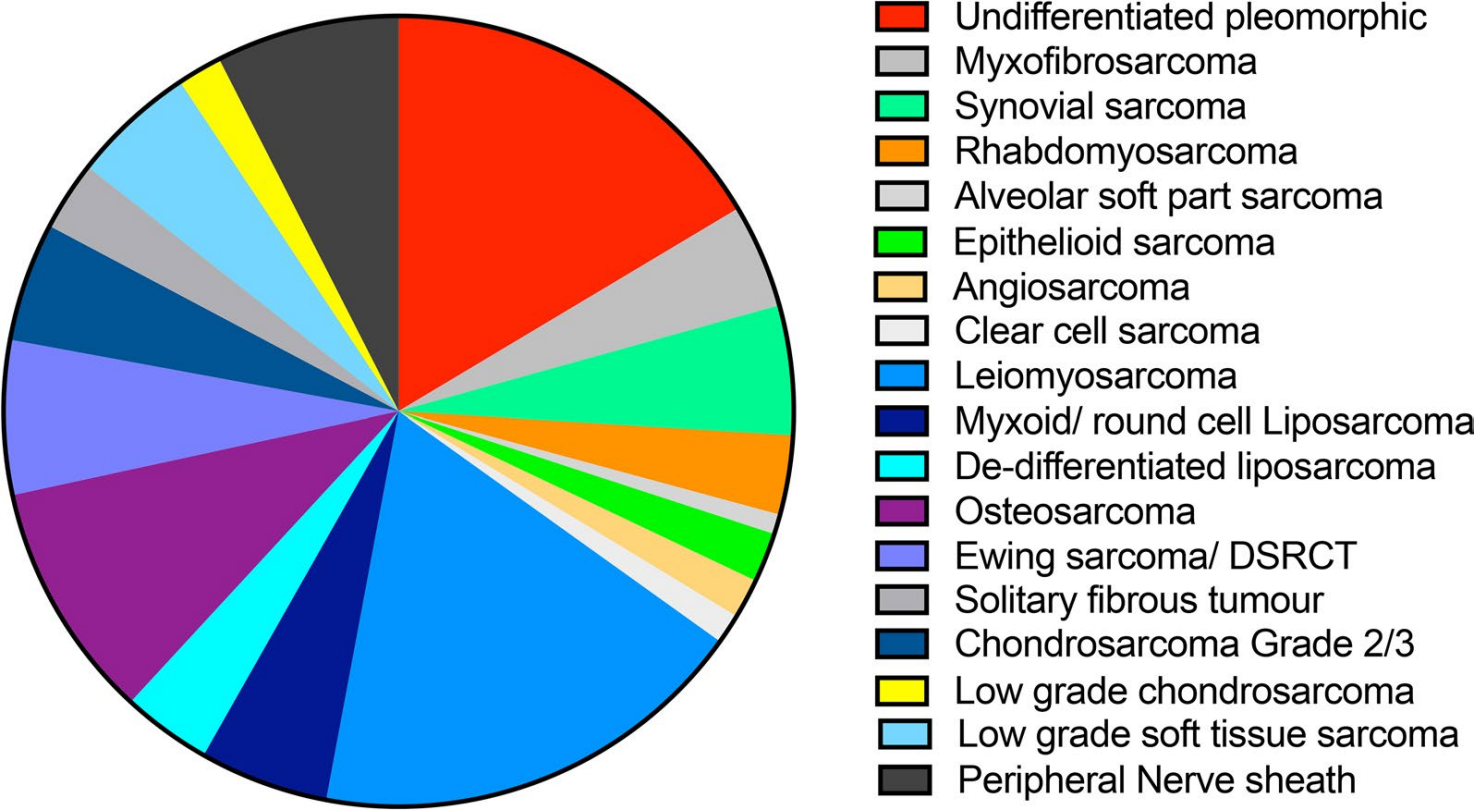
Distribution of molecular-histological subtypes of the 493 sarcoma cases reported that underwent a ^18^F-FDG PET–CT between 2007 and 2014. Following diagnostic biopsy (core needle or excision biopsy), the molecular and histological subtypes of sarcoma were identified. A total of 493 cases of sarcoma were diagnosed and were distributed into the following listed sub-types (minimum 4 cases per subtype, pie chart runs clockwise)

Over the 7-year period of the audit evaluation, a total of 493 patients (age range 15–91, median 55 years) referred to the Oxford MDT with histologically confirmed sarcoma, underwent a total of 957 PET–CT scans during their course of management. The number of patients within sarcoma sub-types, the number of PET–CT studies performed and the clinical indications for performing PET–CT are shown (Table 1, Fig. 1). Most PET–CT scans were performed in high-grade (n = 930) versus low-grade sarcoma (n = 27, e.g. low grade chondrosarcoma and low grade soft tissue sarcoma), with a wide distribution of histological subtypes that reflect the distribution of referred cases (shown are where there were > 4 cases per histological subtype, Fig. 1). PET–CT scans were usually performed as a result of MDT decisions, usually in the context of patients that were being planned to undergo radical treatments, such as radical surgery, and where the scan was for initial staging and exclusion of metastatic disease (36% of scans). All of these cases had concurrent MRI and CT imaging already, mainly for local staging and exclusion of lung metastasis. In this staging population, a further 12% (42/344) then had detectable metastatic disease overall by PET–CT that was not detected by conventional CT/MRI. Re-staging with a new baseline scan occurred in cases with suspected later relapse, prior to potential further radical and salvage treatment (39% of scans), and assessment of treatment response to therapeutics in the remaining (35% of scans, see Table 1).

**Table 1:** Number of patients, PET–CTs and indication for PET–CTs according to sarcoma subtype

Sarcoma pathological diagnosis	Number of patients	Number of PET–CT scans			
		Total	Staging^a^ (with metastasis)	Restaging	Treatment response
Undifferentiated pleomorphic^b^	81	166	64 (6)	62	40
Angiosarcoma^c^	8	11	7 (1)	3	1
Leiomyosarcoma	89	166	43 (8)	84	39
Rhabdomyosarcoma	16	53	13 (4)	15	25
Myxofibrosarcoma	21	30	14 (2)	8	8
Epithelioid sarcoma	10	23	7 (1)	15	1
Osteosarcoma	48	98	39 (7)	28	31
Clear cell sarcoma	6	8	5 (1)	3	0
Ewing sarcoma/DSRCT	31	120	20 (6)	47	53
Synovial sarcoma	26	44	17 (3)	24	3
De-differentiated liposarcoma	18	36	11 (1)	17	8
Solitary fibrous tumour	14	25	8 (1)	9	8
Chondrosarcoma grade 2/3	24	45	18 (1)	17	10
Myxoid/round cell liposarcoma	26	36	18 (4)	12	6
Alveolar soft part sarcoma	4	4	4 (1)	0	0
MPNST	37	65	33 (4)	21	11
Low grade soft tissue sarcoma	25	17	17 (0)	0	0
Chondrosarcoma grade 1	9	10	6 (0)	4	0
Total	493	957	344 (51)	369	244

*DSRCT* desmoplastic small round cell sarcoma, *MPNST* malignant peripheral nerve sheath tumour, includes low-grade

^a^Note this is overall detection of metastatic disease at initial CT and MRI staging only, ‘added value’ comparison is reported in Table 3

^b^Includes high grade ‘spindle cell sarcoma’

^c^Includes ‘high grade epitheloid haemangioendothelioma’

**Fig. 2:**
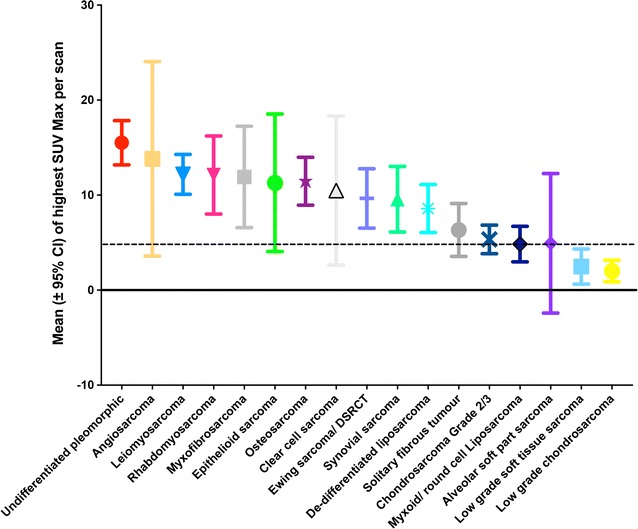
Distribution of the highest SUVmax values per ^18^F-FDG PET–CT from each of 957 scans within each sarcoma diagnostic sub-type. For each PET–CT scan performed in each histological sub-type listed, the highest SUVmax values were collated and distributions determined. A minimum of 4 cases per-subtype. Mean (symbol) and 95% confidence intervals are shown in rank order

**Fig. 3:**
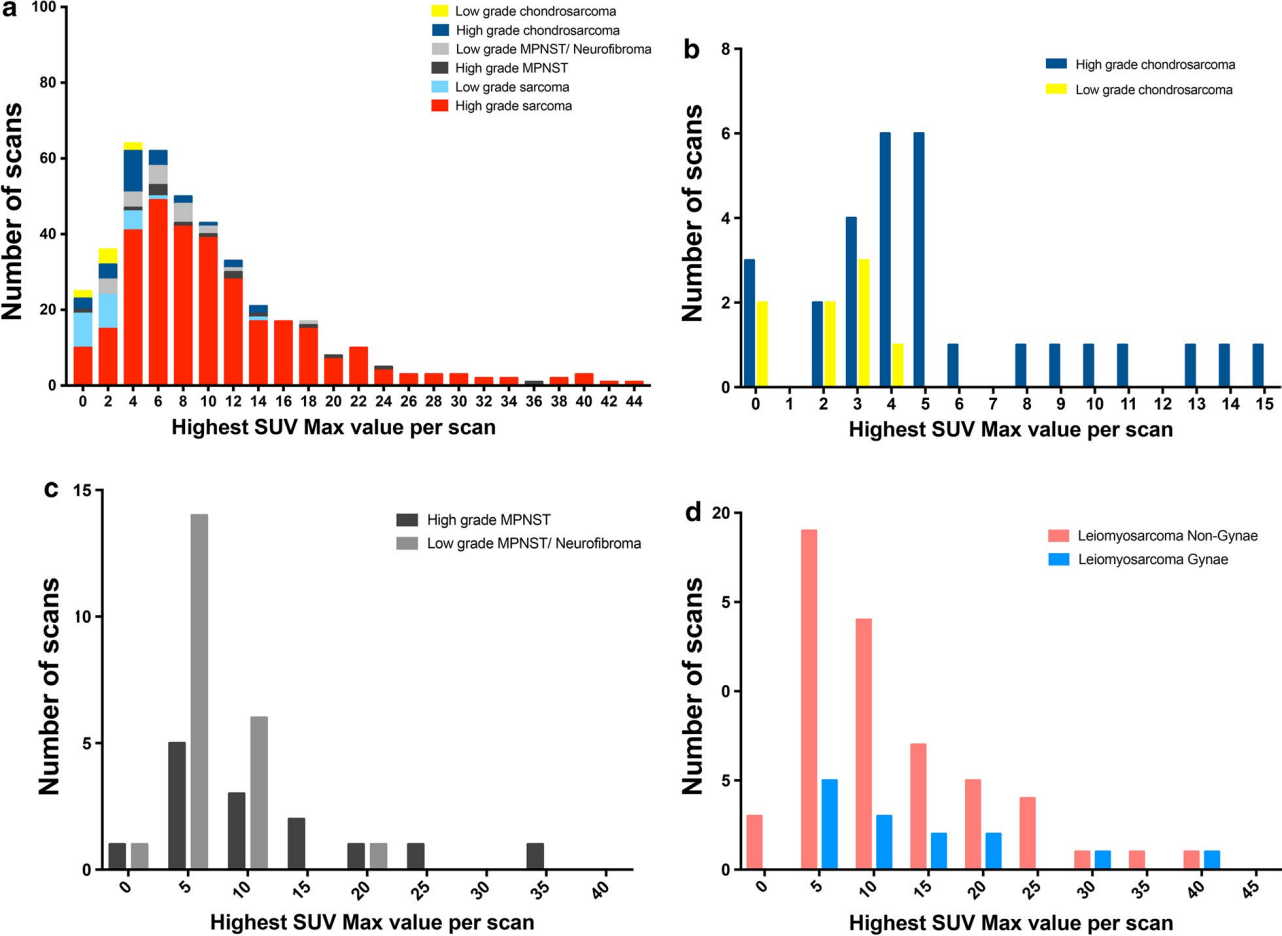
The distributions of the highest SUVmax values per ^18^F-FDG PET–CT scan with respect to examples of sarcoma cases defined histologically as low and high-grade. **a** The highest SUVmax values in each of 957 PET–CT scans are shown with respect to histologically low-or high-grade sarcoma (e.g. low-grade soft tissue sarcoma, chondrosarcoma, MPNST). Note the overlap of SUVmax values between high and low-grade sarcoma in the range of SUVmax values of 4-8 (dashed line at 5). Specific comparison of SUVmax values between; **b** high and low-grade chondrosarcoma, **c** high and low-grade malignant peripheral nerve sheath tumours, and **d** leiomyosarcoma arising from gynaecological (uterine) versus non-gynaecological origin

A statistically significant difference was observed between mean SUVmax of high and of low-grade sarcoma independent of histological subtype (p value < 0.0001, Fig. 2). Most high-grade sarcomas had mean SUVmax values greater than 10, with wide distribution of SUVmax activity rather than a defined cut-off, making the distinction within grade and between histological subtypes more subtle (Fig. 2). Importantly, between the specific low and high-grade subtypes, there appeared significant differences in SUVmax that presumably reflects the differential mechanisms of cellular derangement (Fig. 3a). For the example of chondrosarcoma, the differences between low and high-grade defined histologically also appeared to threshold at the SUVmax value of 4 (Fig. 3b), whereas in MPNST, the spectrum of SUVmax values significantly overlapped depending on the histological classification, making any distinction of grade based on SUVmax less discriminative and less correlative with histology (Fig. 3c). A further factor in relation to SUVmax distribution and histology also includes the anatomical origin of otherwise indentical histologies, exemplified by leiomyosarcoma, with an apparent higher SUVmax observed in gynaecological compared to non-gynaecological (vascular) origin leiomyosarcoma (Fig. 3d).

**Fig. 4:**
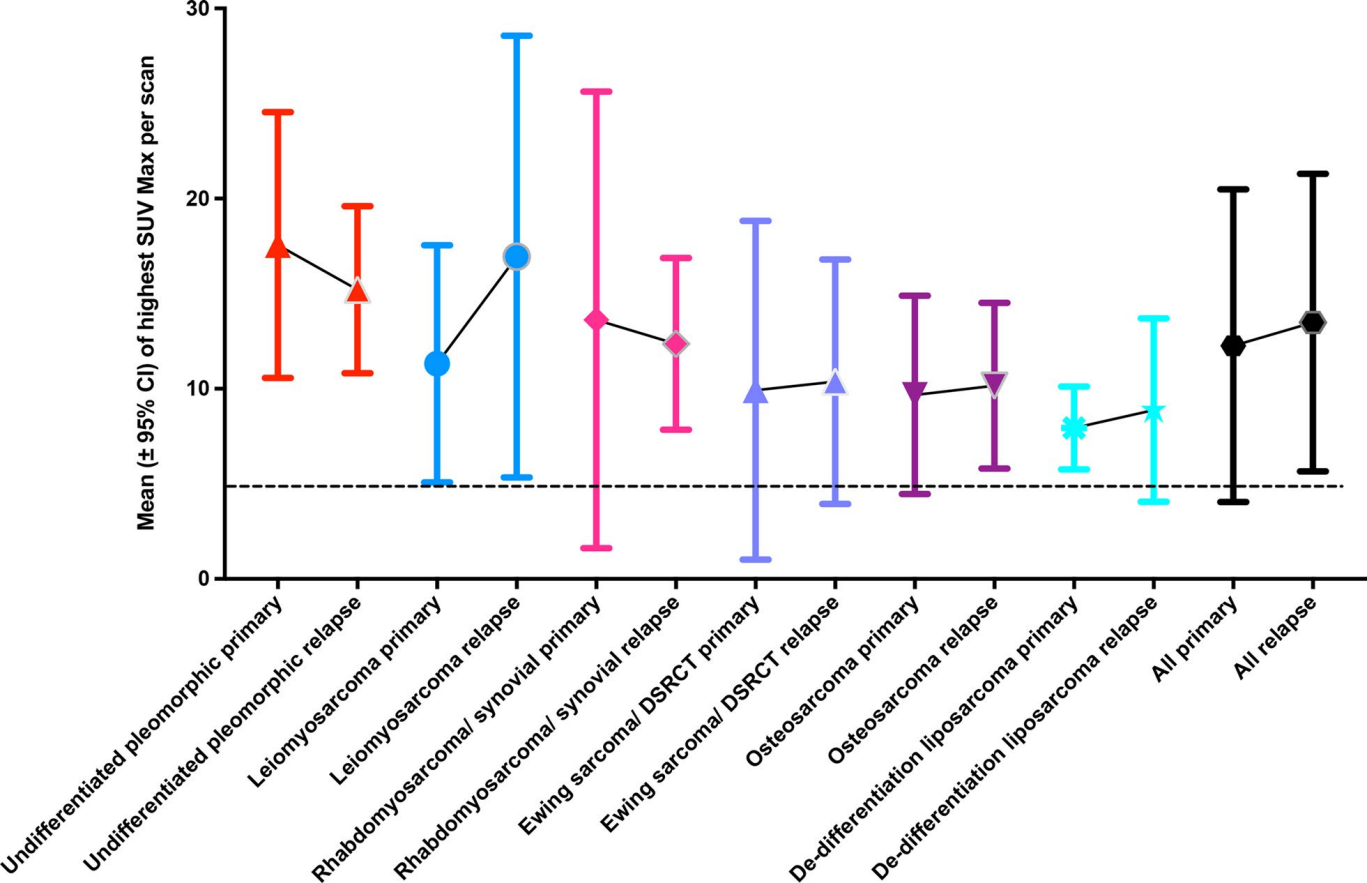
Distribution of the highest SUVmax values from each of 957 ^18^F-FDG PET–CT scans within each sarcoma diagnostic sub-type in the primary lesion and following relapse (re-staging after primary treatment). High-grade sarcoma can have a propensity to relapse after primary (baseline) treatment (e.g. after surgery, radiotherapy and chemotherapy). The figure shows a comparison of the distribution of the highest SUVmax values per scan within subtypes, where there were also SUVmax values at relapse. The mean and 95% confidence intervals show non-significant differences (Non-parametric Mann–Whitney), but are suggestive of potential selection for higher-grade clones (higher SUVmax and altered confidence interval range) in relapse

As a result of intra-tumoural heterogeneity, differences in SUVmax between sarcoma sites within each patient may be manifest at different stages of the disease. SUVmax values were compared between scans performed at primary staging, with those performed later in follow-up in relapse. The expectation was that more aggressive cell types would have populated relapse disease sites compared to the primary tumour, and potentially be reflected in a higher SUVmax in those relapsed sites. Subsequent comparison of the highest SUVmax per scan at primary staging and restaging, grouped by sarcoma histological subtype, revealed no statistically significant differences (Figs. 4), although in some subtypes, trends in SUVmax values may be increasing, e.g. in leiomyosarcoma.

**Fig. 5:**
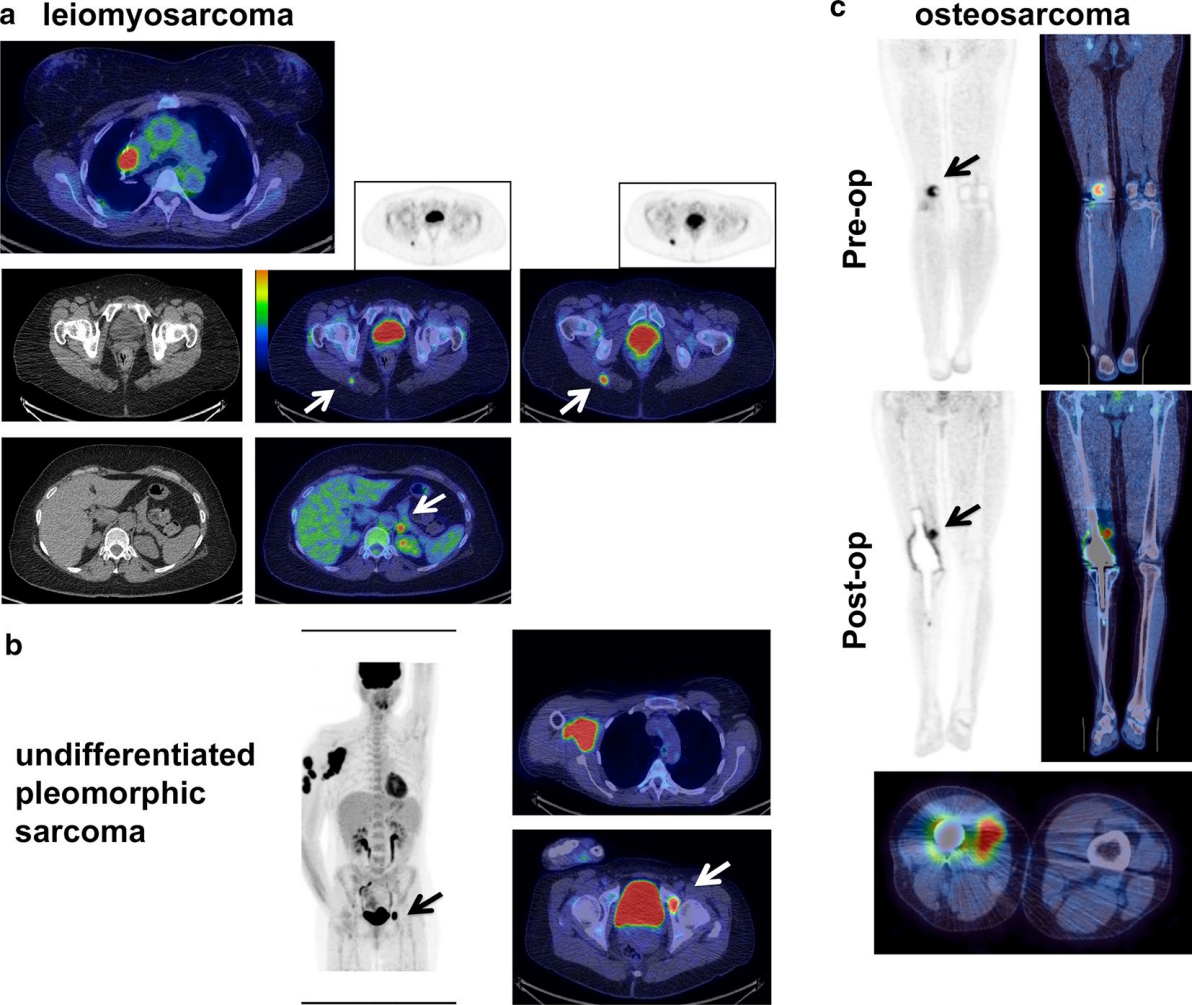
Examples of ^18^F-FDG PET and fused PET–CT images with added value detection of disease sites in sarcoma. All images are on an SUV scale of 0–6. **a** Case of a 57 year-old female with hilar lung metastatic leiomyosarcoma, but with occult metastatic sites (arrows; right buttock and left para-aortic region) not clearly evident on conventional CT scans. **b** Case of a 54 year-old female with undifferentiated pleomorphic sarcoma (UPS) with primary right axillary disease, but with an occult bone secondary in the pelvis (arrow) on PET–CT. **c** Case of a 23 year-old male with distal femur osteosarcoma post MAP chemotherapy (pre-op) and after reconstructive surgery and prosthetic replacement with a local recurrence (post-op). Arrow indicates FDG avid nodule of local recurrence close to the prosthetic margin not visible on CT

Of the 930 PET–CT scans performed in high-grade sarcoma, 193 displayed features considered to have added value over conventional CT and/or MRI performed concurrently (21%, Table 2). Of these 193 PET–CT scans, 56 were performed at initial sarcoma diagnostic staging, 78 at re-staging (new baselines) and 59 in treatment (chemotherapy) responses (Table 2). Here, ‘added value’ refers to any additional specific features offered by PET–CT. Specifically, added value does not only relate to the identification of further metastatic disease sites in addition to those already identified by the conventional CT and MRI imaging, but to those features resulting in so-called ‘upstaging’ of disease. For example, PET–CT specific detection of either occult muscular and soft tissue metastases, small peritoneal or other visceral metastases not visualized by conventional non-contrast enhanced CT (as part of PET–CT) and MRI imaging (Table 3, see examples in Fig. 5). Thus, ‘added value’ of PET–CT reflects detection of metastatic disease (M1) in patients who would be otherwise staged as M0 by conventional imaging approaches. In patients with high-grade sarcoma, it was possible to compare a total of 284 PET–CT scans with the conventional CT and MRI specifically during the initial diagnostic staging. In terms of the presence or absence of metastatic disease (M0 versus M1), of these, 232 patients were true negatives for metastatic disease (negative in both PET–CT and the conventional imaging), 24 were true positive (positive in both PET–CT and the conventional imaging), 23 were false negatives (positively identified disease in PET–CT) and 1 was a false positive (non-disease associated FDG uptake). This retrospective data results in an overall metastatic disease rate of 10% based on conventional CT and MRI, and 22% for detection with dual modality PET–CT. As both CT and PET should both be able to detect the predominant sites of pulmonary metastatic disease, this additional benefit might be expected to be mainly because of detection of non-pulmonary metastatic disease sites. Following scrutiny of these staging PET–CTs, ‘added value’ was indeed associated with detection of occult metastatic sites in bone, muscle and visceral sites, accounting for the PET–CT upstaging (Table 3). Overall, given the prevalence of sarcoma sub-types in this bone and soft tissue sarcoma series, and compared to conventional MRI/CT, PET–CT appeared to have a greater sensitivity (96% vs 54%), positive predictive value (96%) and negative predictive value (99%), even though these sensitivity and specificity values were not prospectively evaluated.

**Table 2:** Number of PET–CTs in which ‘added value’ was detected compared to conventional imaging (MRI/CT) according to sarcoma subtype

Sarcoma pathological diagnosis	Percentage of PET–CT with added value	Number of PET–CT with added value over MRI/CT		
		Staging	Restaging	Treatment response
Undifferentiated pleomorphic^a^	13.3% (22/166)	6	11	5
Angiosarcoma^b^	9% (1/11)	1	0	0
Leiomyosarcoma	18% (30/166)	7	14	9
Rhabdomyosarcoma	24.6% (13/53)	3	2	8
Myxofibrosarcoma	26.7% (8/30)	0	5	3
Epithelioid sarcoma	13% (3/23)	1	2	0
Osteosarcoma	23.5% (23/98)	6	9	8
Clear cell sarcoma	12.5% (1/8)	1	0	0
Ewing sarcoma/DSRCT	27.5% (33/120)	7	12	14
Synovial sarcoma	13.6% (6/44)	3	2	1
De-differentiated liposarcoma	8.3% (3/36)	1	1	1
Solitary fibrous tumour	28% (7/25)	0	5	2
Chondrosarcoma grade 2/3	24.4% (11/45)	3	5	3
Myxoid/round cell liposarcoma	11.1% (4/36)	2	1	1
Alveolar soft part sarcoma	25% (1/4)	1	0	0
MPNST	41.6% (27/65)	14	9	4
Low grade soft tissue sarcoma	0% (0/17)	0	0	0
Chondrosarcoma grade 1	0% (0/10)	0	0	0
Total	21% (193/930)	56	78	59

*DSRCT* desmoplastic small round cell sarcoma, *MPNST* malignant peripheral nerve sheath tumour, includes low-grade

^a^Includes high grade ‘spindle cell sarcoma’

^b^Includes ‘high grade epitheloid haemangioendothelioma’

**Table 3:** Summary of the ‘added value’ features of PET–CT compared to conventional imaging (MRI and CT)

Reasons for added value of PET–CT over conventional imaging	Number of PET–CT scans per indication		
	Staging^b^	Restaging^a^	Treatment response
Occult bone metastases	10	3	1
Recurrence at local site or adjacent to metallic prosthesis not definitive on conventional imaging	0	18	2
Follow up of occult bone metastases	0	8	10
Occult muscular metastases	3	0	0
Follow up of occult muscular metastases	0	3	2
Cardiac metastases (muscular) not detected on conventional imaging	2	1	0
Subcentimetre FDG avid nodes	5	2	1
Static tumoural size, reduced FDG avidity	0	7	16
Static tumoural size, increased FDG avidity	6	2	6
Solitary FDG avid pulmonary nodule, indeterminate on CT	7	3	0
Missed visceral disease on CT/MRI	11	5	5
Metastasis outside the fields of conventional imaging	1	0	0
FDG negative suspected recurrence adjacent to prosthesis or locally in patient with prior markedly avid disease	0	3	2
Intra-lesional heterogeneity—guided biopsy to avoid underestimation of grade	10	6	0
Enlarged FDG negative nodes with moderately FDG avid primary	1	2	0
Increase in tumoural size but reduced avidity	0	0	7
Recurrence at an ablation or surgical site, indeterminate on MRI	0	2	0
Follow up of FDG positive disease adjacent to prosthesis or local surgical site	0	13	7
Total	56	78	59

^a^Restaging = new baseline imaging prior to new clinical management intervention

^b^Actual number of M0 to M1 upstaging = 25

Moreover, ‘added value’ also relates to altered FDG avidity in static sized lesions, either indicating increased SUVmax suggesting disease presence or progression, and decreased SUVmax, as a reflection of responses to oncological treatment. In this context, PET–CT specifically might have ‘added value’ in the characterization of enlarged FDG negative loco-regional lymph nodes that are likely reactive rather than sarcoma containing, and in the detection of FDG avid lesions near prostheses following reconstructive surgery, where MRI and CT artifacts preclude accurate assessment (Table 3). These findings overall are therefore relevant both to achieving accurate TNM staging, but also to directly influence clinical decision-making for subsequent radical surgical, radiotherapy and chemotherapy interventions.

In terms of histological sub-types, the highest overall ‘added value’ appeared in the context of malignant peripheral nerve sheath tumour subtype of sarcoma (41.6%), whereas in most cases of myxofibrosarcoma and solitary fibrous tumour, the advantages of PET–CT were manifest during follow-up re-staging and treatment response assessment (Table 2). For chemo-sensitive sarcoma, ‘added value’ was skewed towards the chemotherapy treatment response assessment scans (e.g. in chemotherapy responsive rhabdomyosarcoma and Ewing sarcoma), and following trabectedin chemotherapy, such as in leiomyosarcoma (Table 2) [21]. For diagnostic staging and re-staging, significant ‘added value’ reasons also included identification of local recurrence sites at and near prostheses (18/138), during the follow up of FDG positive lesions (17/138), for all lesions missed (not easily visible) by conventional imaging (16/138), for intra-lesional heterogeneity to guide diagnostic biopsies (16/138), and for occult (not previously visible) bone metastasis (13/138).

## Discussion

Here we report a retrospective audit and evaluation of PET–CT clinical use in a single tertiary UK sarcoma center. The data represents one of the largest reported series of PET–CT use in routine sarcoma clinical practice, but this does not represent a definitive, prospective, clinical and cost effective evaluation. Despite these caveats, the use of PET–CT in this report does appear in line with evolving guidelines, including the most recent 2016 Royal College of Radiologists, UK guidelines for PET–CT in sarcoma [22]. As such, it provides valuable preliminary evidence on which to base future multi-center evaluation in a routine clinical practice, as a clinical research tool, and in the development of evidence suitable for supporting appropriate reimbursement.

In sarcoma, oncogenic drivers frequently result in up-regulation of glucose transporters, such as GLUT4 [23], resulting in higher ^18^FDG SUVmax levels associated with aggressive cell behavior, and when there is associated immune cell infiltration, such as in giant cell tumour of bone [24]. As expected, we observed statistically significant differences of mean SUVmax between low-grade and high-grade sarcoma histological sub-types. Moreover, significant variation in SUVmax was observed between different patients who had the same sarcoma sub-type. The latter probably reflects the functional heterogeneity within the tumour and its associated micro-environment, indicating that between sarcoma subtypes, primary and metastatic disease, there are differences in SUVmax that reflect those at the molecular, cellular and micro-environment level. PET–CT appeared useful in identifying sarcoma sites that contained both high and low- grade elements (the extremes of heterogeneity), for example in de-differentiated liposarcoma, de-differentiated chondrosarcoma and in MPNST. SUVmax values > 5 were consistent with higher-grade disease, except in the case of MPNST, where there was much less correlation of SUVmax with histological assessment of grade. Thus, low-grade soft tissue sarcoma and low-grade bone sarcoma (e.g. chondrosarcoma) do not probably warrant routine PET–CT estaging valuation, unless there is clinical suspicion of high-grade transformation, such as in either large axial or pelvic chondrosarcoma.

The additional benefits (“added value”) of PET–CT appeared to be in a range of clinical contexts, making specific comparisons and overall evaluation more complex. Here we attempted to simply compare conventional CT and MRI scans performed concurrently with the PET–CT, in order to scope the scenarios that might justify PET–CT as a routine dual scanning modality, and that impacts on potentially clinically important features. What we have not been able to fully assess in all circumstances, is to prospectively prove that direct and important changes to subsequent clinical management of patients ensued as a result of the PET–CT results, and the PET–CT results alone. For example, there can be no doubt from the radiological and clinical point of view, that the ‘added value’ of PET–CT in upstaging of disease from M0 to M1 would have had clinical impact that might make the difference between either reconstructing a limb, resecting a tumour or amputation. In this regard, our evidence suggests that whole body PET–CT has particular utility in detection of occult non-pulmonary disease not visible on conventional CT and MRI. As both CT and PET should both be able to detect the predominant sites of pulmonary metastatic disease, scrutiny of these staging PET–CTs for ‘added value’ was indeed associated with detection of occult metastatic sites in bone, muscle and visceral sites, accounting for the PET–CT upstaging. Moreover, ‘added value’ to guiding biopsies to active and viable areas of the tumour, thereby avoiding regions of necrosis, and in the post-operative setting, where MRI artifacts near metallic prostheses prevents adequate visualization of potential local recurrence, are also bona fide reasons to adopt PET–CT [8, 25, 26]. Conversely, inflammation related ^18^FDG avid foci in PET–CT studies immediately following surgery, chemotherapy and radiotherapy, can be mistaken for tumour deposits, and can be miss-interpreted by inexperienced reporting, resulting in false positives.

The choice of imaging is therefore a significant consideration when determining the timing of assessment, and may bias the subsequent reporting of results in this study. In the treatment response assessment, PET–CT addresses the discrepancy between the change in lesion size and metabolic response, and was the commonest reason for “added value” of PET–CT over conventional imaging in our series. In the staging and re-staging groups, occult metastatic disease and FDG avid nodal disease, which were not enlarged by CT criteria, may have led to improved TNM staging accuracy overall. Likewise, the presence of new sites of disease (occult disease) in the standardized dimension assessment of oncological treatment response, also indicates the need to stop current treatment and to re-evaluate alternative strategies. Whilst the staging and later findings related to ‘added value’ are based on comparison of imaging modalities performed within a few weeks, and whilst some sarcomas may rapidly grow over such a time frame, these cases are very rare, and so we believe that the comparison period is valid.

The heterogeneity of the behavior of sarcoma cells points to the potential for high-grade cell types with more aggressive features to populate recurrent sites of disease detected at restaging. SUVmax alone does not, however, constitute a complete analysis of heterogeneity, and this question requires further analyses, as the overall ^18^FDG distribution within tumours has not been assessed. Further prospective validation of PET–CT in sarcoma is justified, as ‘added value’ components may also reflect differences in reporting experience and protocols. To eliminate future bias, the prospective evaluation of conventional imaging compared with PET–CT across institutions will require standardized reporting (independent double blind reporting) for both modalities. Moreover, quantification of patient outcomes with respect to changes in clinical management would need to be prospectively compared, as well as staging accuracy and cost effectiveness for the patient pathway. In summary, PET–CT in high-grade sarcoma offers the prospect additional benefit in routine staging of high-grade sarcoma at baseline, and specific staging situations during relapse and treatment.

## Conclusion

We report a large retrospective audit of PET–CT in bone and soft tissue sarcoma with varied grade in a single multi-disciplinary centre. A total of 957 consecutive PET–CT scans were performed in a single supra-regional centre in 493 sarcoma patients (excluding GIST) between 2007 and 2014. High-grade (II/III) bone and soft tissue sarcoma correlated with high SUVmax, especially undifferentiated pleomorphic sarcoma, leiomyosarcoma, translocation induced sarcomas (Ewing, synovial, alveolar rhabdomyosarcoma), de-differentiated liposarcoma and osteosarcoma. We identified added utility of PET–CT in addition to MRI and CT in high-grade sarcoma of bone and soft tissues. An estimated 21% overall potential benefit was observed for PET–CT over CT/MRI, and in particular, in ‘upstaging’ of high-grade disease (from M0 to M1) where an additional 12% of cases were deemed M1 following PET–CT. This large study suggests PET–CT in high-grade bone and soft tissue sarcoma can add significant benefit to routine CT/MRI staging. Further prospective and multi-centre evaluation of PET–CT is warranted to determine the actual predictive value and cost-effectiveness of PET–CT in directing clinical management of clinically complex and heterogeneous high-grade sarcomas.
